# Avoiding False Positive Antigen Detection by Flow Cytometry on Blood Cell Derived Microparticles: The Importance of an Appropriate Negative Control

**DOI:** 10.1371/journal.pone.0127209

**Published:** 2015-05-15

**Authors:** Emerence Crompot, Michael Van Damme, Hugues Duvillier, Karlien Pieters, Marjorie Vermeesch, David Perez-Morga, Nathalie Meuleman, Philippe Mineur, Dominique Bron, Laurence Lagneaux, Basile Stamatopoulos

**Affiliations:** 1 Laboratory of Clinical Cell Therapy, Université Libre de Bruxelles (ULB), Jules Bordet Institute, Brussels, Belgium; 2 Hematology Department, Jules Bordet Institute, Brussels, Belgium; 3 Laboratory of Molecular Parasitology, IBMM, Université Libre de Bruxelles (ULB), Gosselies, Belgium; 4 Department of Hemato-Oncology, Grand Hôpital de Charleroi, Gilly, Belgium; INSERM, FRANCE

## Abstract

**Background:**

Microparticles (MPs), also called microvesicles (MVs) are plasma membrane-derived fragments with sizes ranging from 0.1 to 1μm. Characterization of these MPs is often performed by flow cytometry but there is no consensus on the appropriate negative control to use that can lead to false positive results.

**Materials and Methods:**

We analyzed MPs from platelets, B-cells, T-cells, NK-cells, monocytes, and chronic lymphocytic leukemia (CLL) B-cells. Cells were purified by positive magnetic-separation and cultured for 48h. Cells and MPs were characterized using the following monoclonal antibodies (CD19,20 for B-cells, CD3,8,5,27 for T-cells, CD16,56 for NK-cells, CD14,11c for monocytes, CD41,61 for platelets). Isolated MPs were stained with annexin-V-FITC and gated between 300nm and 900nm. The latex bead technique was then performed for easy detection of MPs. Samples were analyzed by Transmission (TEM) and Scanning Electron microscopy (SEM).

**Results:**

Annexin-V positive events within a gate of 300-900nm were detected and defined as MPs. Our results confirmed that the characteristic antigens CD41/CD61 were found on platelet-derived-MPs validating our technique. However, for MPs derived from other cell types, we were unable to detect any antigen, although they were clearly expressed on the MP-producing cells in the contrary of several data published in the literature. Using the latex bead technique, we confirmed detection of CD41,61. However, the apparent expression of other antigens (already deemed positive in several studies) was determined to be false positive, indicated by negative controls (same labeling was used on MPs from different origins).

**Conclusion:**

We observed that mother cell antigens were not always detected on corresponding MPs by direct flow cytometry or latex bead cytometry. Our data highlighted that false positive results could be generated due to antibody aspecificity and that phenotypic characterization of MPs is a difficult field requiring the use of several negative controls.

## Introduction

In recent years, a large number of publications have established that cells are able to produce ‘‘extracellular vesicles” (EVs), which are important mediators of physiological processes in normal and pathological cells (e.g., cell growth, activation, proliferation, apoptosis, senescence) [1;2]. EVs principally include three populations distinguishable by size, composition and biogenesis: exosomes (50–100 nm in diameter), microparticles (100 nm to 1 μm) and apoptotic bodies (AB; 1 μm to 4 μm) [3]. In this study, we focused on microparticles (MPs), also called microvesicles (MVs) by some authors. These particles are released into the extracellular space by outward budding and fission of the plasma membrane [4–6]. The release of vesicles is efficiently induced upon cellular activation or apoptosis and the subsequent increase of intracellular Ca2+. These MPs contain proteins and nucleic acids, including cytoplasmic and membrane proteins [7], mRNAs [8;9], microRNAs (miRNAs) [10–12], non-coding RNAs (ncRNAs) [13], and DNA [14–17]. All of these elements can be delivered to other cells by different mechanisms [4;18]. MPs normally feature antigens from parental cells and phosphatidylserine (PS), which can be detected by annexin-V staining [19;20]. However, some observations also suggest the existence of MPs without PS externalization [21–25].

The characterization of MPs is most often performed by flow cytometry, which is considered the gold standard technique used in 75% of MP publications. Lacroix et al defined an accurate MP gate between 0.3 and 1 μm as the best compromise between good resolution and a level of background noise that does not impede cytometer performance [26]. Over the years, other techniques have been applied to improve the study of MPs such as electron microscopy, ELISA, nanoparticle tracking analysis, and atomic force microscopy [27].

The field of MP study is rapidly expanding. It has been already shown that MPs in body fluids could be used as prognostic markers for pathologies that include cardiovascular diseases, inflammation, sepsis, lupus, HIV, and several cancers [28–31]. MPs also have significant potential for clinical applications, especially in brain cancer, where EVs have been used as delivery vehicle to transport therapeutic molecules [32–34].

However, some discrepancies exist in literature concerning phenotypic characterization of MPs. Ghosh et al [35] and Macey et al [36] were able to detect some CD19+ B lymphocyte-derived MPs, Blanchard et al [37] showed CD3+ T lymphocyte-derived MVs while Miguet et al [38] demonstrated by proteomic study that these antigens were not found in vesicles. Blanchard et al highlighted also that CD28, CD40L and CD45 were not found on MVs derived from T lymphocytes despite these antigens were clearly detected in the original cells [37]. In addition, since MP analysis by flow cytometry is quite difficult due to their small size, several authors [37;39;40] used technique based on latex beads with different protocols. These beads can generate non-specific staining depending on the choice of antibody, or saturation methods and thus false positive results. In the present paper, we demonstrated that several results published in the literature are more than probably wrong due to the use of inappropriate controls. The purpose of this study was thus to clarify antigen detection on MPs from blood cells and to propose new negative control in MP analysis by flow cytometry to avoid false positive results.

## Materials and Methods

### Ethics statement

This study has been approved by the Bordet Institute Ethics Committee and conducted according to the principles expressed in the Declaration of Helsinki. All samples were collected after written informed consent.

### Biological samples and cell culture

Peripheral blood mononuclear cells (PBMC) were obtained from healthy donor buffy coat (provided by the “service francophone du sang de la croix rouge de Belgique”). PBMC were isolated by Ficoll-Hypaque gradient centrifugation as previously described [41]. B-cells, T-cells, NK-cells, and monocytes were purified by positive selection, using CD19, CD3, CD56, and CD14 microbeads, respectively (Macs Miltenyi Biotec, Leiden, the Netherlands). The purity of cells was from 97.46 to 99% after immune magnetic enrichment (data not shown). Platelets were obtained from expired single-donor platelet apheresis units. They were collected by the “service francophone du sang de la croix rouge de Belgique”. Chronic Lymphocytic Leukemia (CLL) samples were obtained from CLL patients with informed written consent. The normal purified cells and CLL B-cells were cultured at densities between 4–70 x 10^6^ cells/ml (according to cell type) in 0.2 μm filtered RPMI 1640 (Lonza, Basel, Switzerland). A serum-free medium was used to avoid contamination by Fetal Bovine Serum (FBS)-derived vesicles [42]. We also used bone marrow mesenchymal stromal cells (BM-MSCs) to include a non-hematopoietic negative control. BM-MSCs were harvested from the sternum or iliac crest of healthy volunteers and were isolated by the classical adhesion method, as previously described [43]. BM-MSCs were plated at 1000 cells/cm^2^ and cultures were used at a subconfluent state (2 x 10^6^ cells). BM-MSCs were cultured in a T-175 flask with serum-free DMEM (Dulbecco’s modified Eagle medium-low glucose) filtered through a 0.2 μm filter. Cells were incubated at 37°C in a 5% CO_2_ humidified atmosphere. With the exception of platelets, all samples were cultured for 2 days prior to MP isolation. Cell viability was analyzed by Trypan blue exclusion assay and confirmed by Annexin V/7AAD labeling (S1 Table).

### Microparticle isolation

MPs were prepared from the supernatant of several cell cultures (healthy purified cells, CLL cells, and BM-MSCs). Although a standardized centrifugation protocol has not been established, a majority of authors have used a 20,000 x g centrifugation for MP recovery, while a 100,000 to 200,000 x g centrifugation is generally necessary to isolate exosomes [26;44–46]. Cell-free supernatant (1ml of blood cell culture and 50ml of MSC culture) was obtained by 2 successive centrifugations at 300 x g for 10 minutes (for platelet samples, centrifugation at 450 x g for 15 minutes was used to remove a majority of platelets). The supernatants were then subjected to 20,000 x g centrifugation for 1 h at 4°C (Ultracentrifuge MX 120+, Swinging Bucket rotor S50-ST, k-factor 77). The MP pellet was washed using 0.2 μm filtered phosphate buffered saline (PBS) and MPs were again centrifuged for 1 h at 20, 000 x g. The MP pellet was finally reconstituted in 150 μl of PBS and stored at -80°C. To ensure that the freezing step do not affect the labeling results, we performed a staining comparison between the samples, before and after the congelation (data not shown).

### Antibodies and other reagents

Because unfiltered buffers and antibodies have been shown to contain interfering elements that can give false positive signals in cytometry [47–51], all products used here were filtered through 0.2 μm filters (VWR, Leuven, Belgium). The following monoclonal antibodies were used: CD19-PE (LT19), CD3-PE(BW264/56), and CD14-PE(TÜK4), CD61-PE(Y2/51) (Macs Miltenyi). CD19-PC5(HIB19), CD20-PE(2H7), CD11c-PE(B-ly6) (BD Pharmingen, Erembodegen, Belgium), CD16-PE(3G8), CD41-PE(P2), CD56-PC5(HLDA6), CD5-PE(BL1a)(Beckman Coulter, Marseille, France), CD8-PC5(DK25) (Dako, Heverlee, Belgium) and CD27-Percp(0323) (Biolegend, San Diego, CA, USA). Annexin-V (Invitrogen) and 7-AAD (BD Pharmingen) were applied to detect apoptotic bodies. Appropriate PE, PE-Cy5, APC isotypes were used as negative controls, all from BD Pharmingen. All the concentrations/clones/origins were reported in S2 Table. Each couple of antibodies were tested in PBS alone to prove their perfect matching, the geometric means were similar between specific antibodies and their isotype control, for all the tested antibodies (data not shown).

### Cell and MP flow cytometry analysis

All analyses were performed on a Navios cytometer (Beckman Coulter). The MP gate was established based on light scattering and size properties (Forward scatter- FSC W2), using Megamix Plus-FSC beads of 0.1, 0.3, 0.5, and 0.9 μm (Biocytex, Marseille, France) and defining MPs as events <1 μm. The lower detection limit was defined as a threshold above the electronic noise of the flow cytometer (0.3 μm). Annexin-V-FITC was used as a general marker for MPs [25;52]. Both annexin-V positive and negative MPs were analyzed in all experiments. Cells and MPs were characterized for the expression of following antigens: CD19 and 20 for B-cells, CD3, 8, 5, and 27 for T-cells, CD16 and 56 for NK-cells, CD14 and 11c for monocytes, CD41 and 61 for platelets. A total of 10 μl of isolated MPs were stained for 15 minutes at room temperature in the dark with 10 μl of annexin-V-FITC and 10 μl of specific antibody. The samples were previously diluted with 20 μl of PBS and 50 μl specific buffer for annexin-V binding (Invitrogen). We performed serial dilutions of antibodies to determine the optimal antibody dilution and to optimize the separation of positive and negative signals. We also used different fluorochromes and isotype controls to confirm our previous results (S3 and S4 Tables). The CD3 titration on T-cells and T-cell derived MPs and CD41 on platelet derived MPs are shown on S1 and S2 Figs.

Events were acquired during 2 minutes of medium flow. Lysis with 0.05% triton was used to monitor false positive signals caused by protein complex (PC); MPs, but not PCs, have been shown to be lysed by triton [53]. To ensure that we analyzed MPs only, we labeled samples with annexin-V and 7-AAD to detect the possible presence of AB. The same labeling was realized without Ca^2+^ binding and without 7AAD as a negative control.

For cell immunophenotypic analysis, all cells were washed in PBS, suspended in 100 μl and labeled with a cell-specific antibody (10 μl) for 15 minutes in the dark. Cells were washed with PBS and a minimum of 20,000 cells were acquired. Data collected from all experiments were analyzed using FSC 3.0 (De novo analysis software, Los Angeles, CA, USA).

### Characterization of MPs by the latex bead technique

Different protocols utilizing latex beads can be found in the literature [37;54;55]. We first used the method described by Wu et al and Mokarizadeh et al [39;40], but we obtained false positive results when MP-coated beads were incubated with antibodies despite the use of a blocking solution such as Bovine Serum Albumin (BSA), Fetal Calf Serum (FCS) or glycine solution, confirming the observations made by Oksvold et al. [56]. Protein concentration was determined with a Nanodrop (Thermo scientific, Nanodrop 2000c)[57]. A total of 15 μg (60 μg for platelets) of MPs in 100 μl of PBS were incubated with 10 μl of antibody for 15 minutes in the dark. Samples were washed with PBS and centrifuged at 20,000 x g for 1 hour at 4°C. Labeled MPs were resuspended in 1 ml of PBS and 1 μl of beads (aldehyde sulfate latex beads 4 μm-Invitrogen) was added. Samples were incubated overnight at 4°C under gentle agitation, followed by a wash step with centrifugation at 300 x g for 10 minutes. Beads were also incubated with the antibody ‘‘washed at 20,000 x g” (without MPs) as the negative control. Labeling of MPs with a negative marker (a marker that is not present on the cells from which the MPs were derived; for example, CD19 for CD3-derived MPs) before incubation with beads was used as a second negative control. The coated beads were resuspended in PBS before reading with a MACSQuant analyzer (Miltenyi Biotec). All data were analyzed using FSC 3.0 (De novo analysis software, Los Angeles, CA, USA).

### Transmission and scanning electron microscopy

Electron microscopy was used to study the morphology of MPs. The MP pellets obtained by centrifugation were submitted for TEM (transmission electron microscopy) and SEM (scanning electron microscopy). For TEM, 40 μl of vesicle suspension were placed on a carbon-coated EM grid, and 0.4 μl of 25% glutaraldehyde was added. Vesicles were then allowed to settle onto the grid overnight at 4°C. Grids were then blotted on filter paper and stained for 30 seconds with 2% uranyl acetate. After further blotting and drying, samples were directly observed on a Tecnai 10 TEM (FEI). Images were captured with a Veleta camera and processed with iTEM and Adobe Photoshop software. For SEM, samples were fixed overnight at 4°C in 2.5% glutaraldehyde, 0.1 M cacodylate buffer (pH 7.2); placed on a glass coverslip; and incubated overnight at 4°C to allow the vesicles to settle. Samples were then rinsed and post-fixed in 2% OsO_4_ for 1 h. After serial dehydration in ethanol, the samples were critical-point dried and coated with platinum according to standard procedures. Imaging was carried out on a Tecnai FEG ESEM Quanta 200 (FEI), and images were processed with iTEM and Adobe Photoshop. We also performed immunogold labeling to confirm our data with a more sensitive technique to detect antigen on MPs. For this assays, purified MPs were fixed in paraformaldehyde 2% as previously described by C. Thery et al. [58]. Grids containing the samples were blocked with PBS with 10% FBS (previously ultracentrifuged during 18h [59]). Each antibody was diluted in PBS with 5% FBS to obtain a concentration of 5μg/ml. Antibody (CD5) was incubated with MPs during 30 minutes before 3 washing. The grids were then incubated with gold-labeled secondary antibodies (goat secondary antibody to mouse IgG 10nm gold /Abcam, Cambridge, England) diluted in PBS with 2% FBS for 30 min, and then washed 3 times. The grids were then observed under electron microscope. These data were added in S3 Fig.

## Results

### Detection of MPs by flow cytometry and electron microscopy

**Fig 1:**
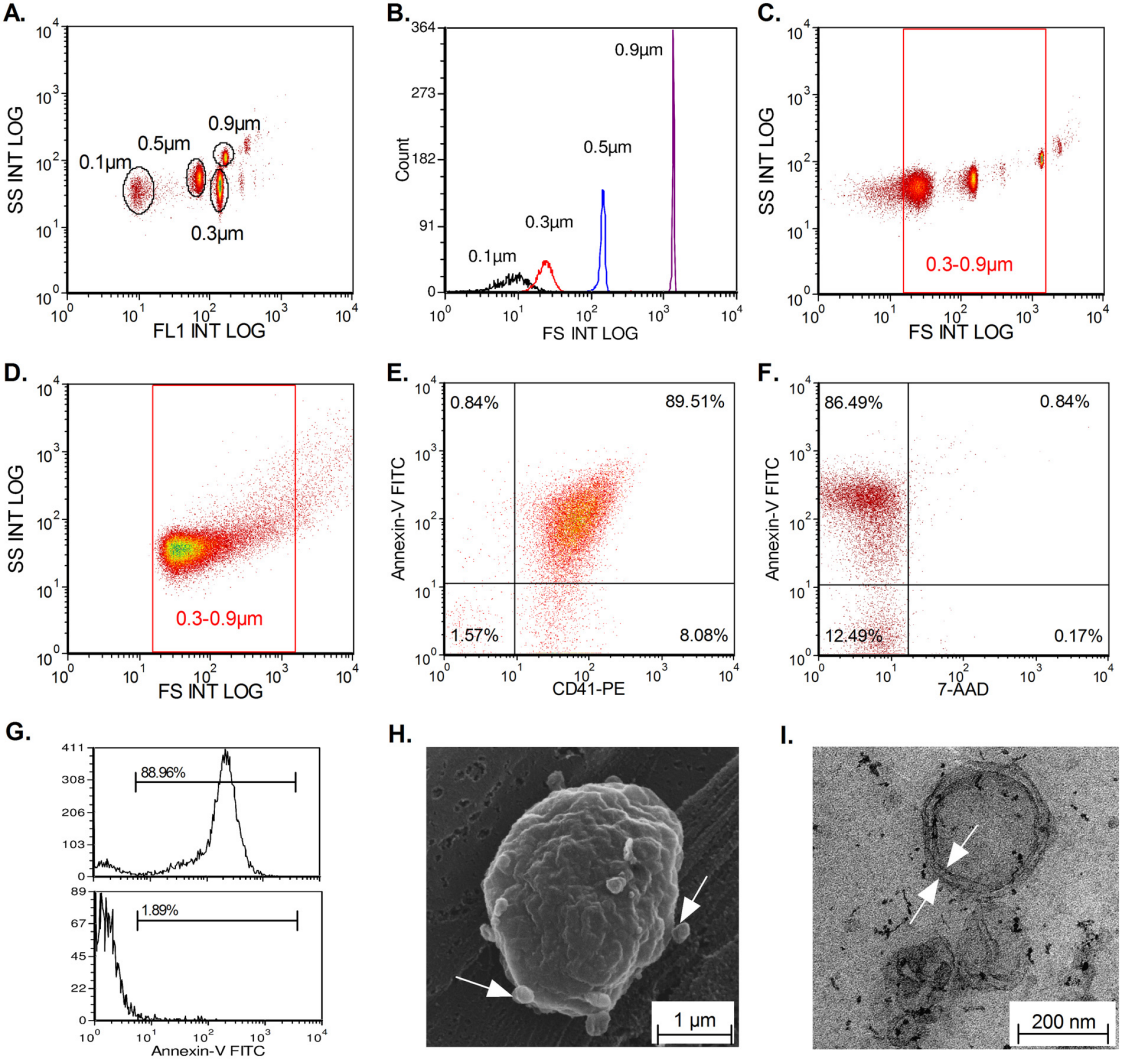
MP analysis by flow cytometry and electron microscopy. Calibration of Navios cytometer using 0.1, 0.3, 0.5, and 0.9 μm beads (Biocytex). The cytometer is able to differentiate the 4 different populations of beads (A-B). We can delimit the gate in accordance with size to study MPs (0.3–0.9 μm) (C). Characteristic elongated shape of MPs repartition limited in a gate of 0.3–0.9 μm. (D). Labeling of platelet derived-MPs with CD41 and annexin-V revealed a double positive population for these markers (E). Example of 7-AAD/annexin V labeling to detect the presence of apoptotic bodies (AB). (F). Absence of contamination with AB. To monitor the presence of protein complexes (PC), lysis with 0.05% triton was used. After lysis, the positive signal disappeared (G). Scanning Electron Microscopy (SEM) shows the production of MPs by B-lymphocytes (white arrows) (H). Transmission electron microscopy (TEM) shows the structure of one MP with a characteristic lipid bilayer (white arrows) (I).

To characterize MPs, a Navios cytometer (Beckman Coulter, Marseille, France), an FSC optimized instrument, was used. As shown in Fig 1, the cytometer was calibrated using a standardized protocol from Biocytex. Briefly, 0.1 μm, 0.3 μm, 0.5 μm, and 0.9 μm Megamix beads-FCS plus were detected in channel FL1 (Fig 1A). With the forward scattering resolution of this cytometer, we were able to accurately distinguish the different bead sizes (Fig 1B), which allowed to define a standardized size-related gate for MP analysis (Fig 1C). Fig 1D shows a representative scatter plot of an MP population from PBMC. We also observed that the population of smaller MPs was more significant than the population of larger MPs. Double labeling with annexin-V-FITC and CD41-PE allowed targeting of platelet derived-MPs (Fig 1E). Although apoptotic bodies (AB) were excluded by size criteria, we monitored for their possible presence by double staining with annexin V and 7AAD. As shown in Fig 1F, AB were not detected in our samples. The presence of protein complex (PC) can also interfere with MP analysis; therefore, we used lysis with 0.05% triton to evaluate the proportion of PC, which we found was <2% in all analyzed samples (Fig 1G). Finally, MPs were analyzed by electron microscopy. Fig 1H shows the production of MPs by B-lymphocytes by scanning electron microscopy. Furthermore, the presence of MPs in our culture supernatant was demonstrated using TEM, which revealed the characteristic spherical vesicle with a lipid bilayer (Fig 1I).

### Immunophenotypic characterization of blood cells and blood cell-derived MPs by direct flow cytometry

**Fig 2:**
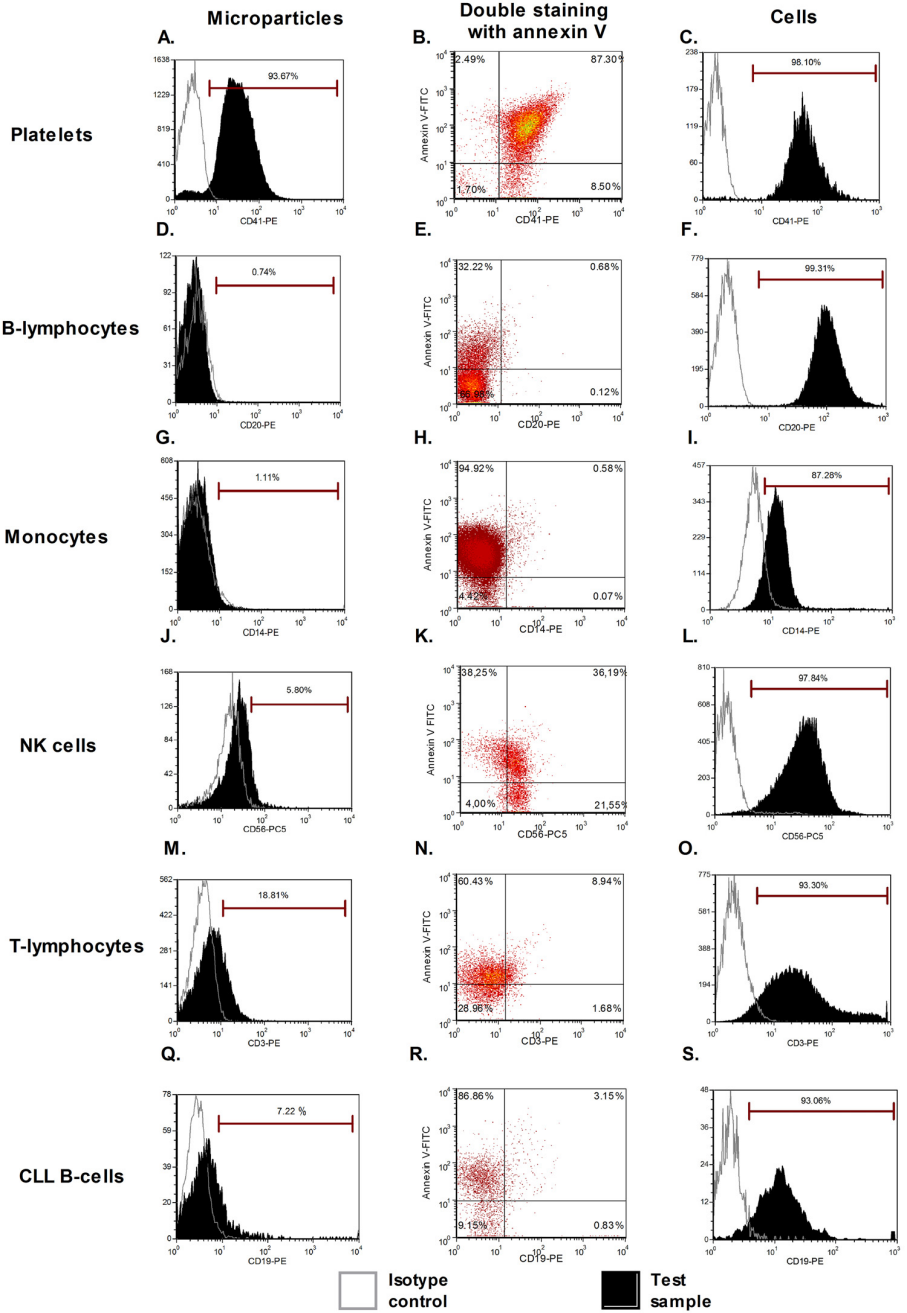
MP characterization by direct flow cytometry. A representative case for each staining is provided: for platelet-derived MPs, CD41 (A) associated with annexin-V labeling (B), similar to that found in the original cells (C). For MPs derived from other cellular sources, no antigen was detected (CD20 and CD14) (D/G) despite the expression of these markers on cells (F/I). An annexin-V positive population is present for all samples (ranging from 32% to 94%). For NK-cells, T-lymphocytes, and CLL B-cells, we observed a weak positive signal with CD56, CD3, and CD19, respectively. These signals were thus false positives, demonstrated by the use of other negative controls (Fig 3). Isotypes were used as negative controls (open line) and the tests are presented by filled histogram.

**Fig 3:**
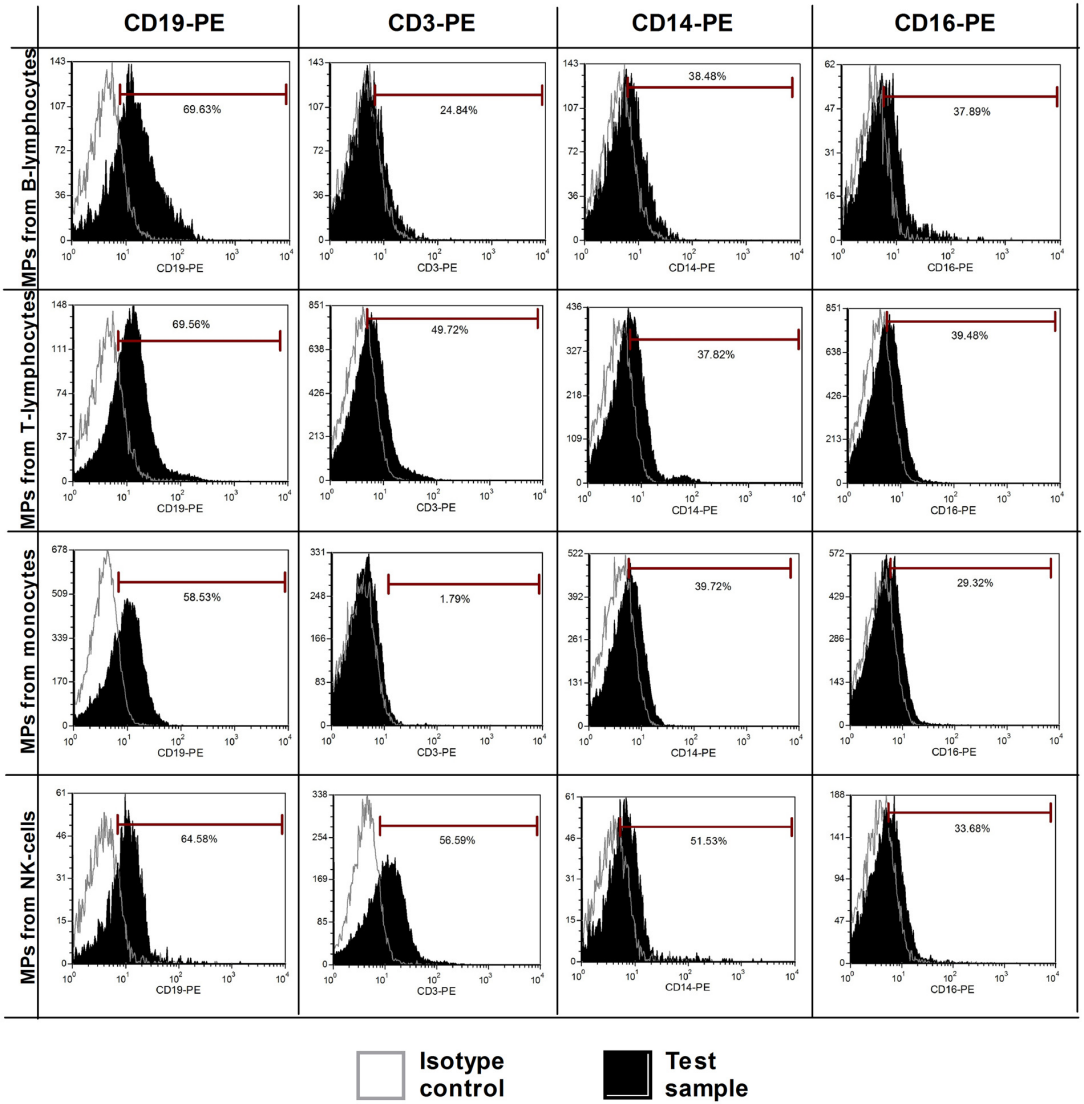
Cross-labeling MPs from different cell types: non-specific labeling. Double staining with annexin-V and specific markers was applied to four MP types (B-lymphocytes, T-lymphocytes, monocytes and NK-cells). Isotypes were used as negative controls (open line) and the tests are presented by filled histogram. Histograms of cell-derived MPs stained with CD19, CD3, CD14, and CD16 showed that non-specific labeling occurs for the majority of samples.

We first investigated the expression of several antigens on the cell surface and cell-derived MPs by direct flow cytometry (n = 5). Signals from platelet markers (CD41 and CD61) were clearly positive on both platelet-derived MPs and platelets (Fig 2A–2C). These initial results indicated that we were able to detect annexin V+/marker+ MPs. These first 2 antigens were largely described in the MP literature and were used as positive controls to initialize the setup of our experiments. However, for all other cell-derived MPs (from B-cells, T-cells, NK cells, monocytes, and CLL B-cells), the markers used were not detected, despite the fact that these antigens were highly expressed by the source cells. A representative example of some of these markers is provided in Fig 2. For CD56, CD3, and CD19 staining, a weak positive signal was observed compared to the isotopic control (Fig 2J, 2M and 2Q). However, this signal was a false positive, shown by using the same antibodies to label MPs from different origins (Fig 3). It should be noted that CD14 labeling on monocytes was quite weak, most likely due to the positive selection with anti-CD14 beads (Fig 2I). Heterogeneous expression of phosphatidylserine dependent on the cellular source of MPs was also observed. Double staining with annexin-V-FITC and specific markers was applied on four MP types (B-lymphocytes, T-lymphocytes, monocytes, NK-cells) with cross-labeling different MPs to detect false positive expression (Fig 3). Histograms of these cell derived-MPs stained with CD19-PE, CD3-PE, CD14-PE, and CD16-PE showed that non-specific labeling occurred in a majority of samples. Means of the Mean Fluorescence Intensity Ratio (MFIR) for all antibodies (studied on both annexin-V positive and negative MPs, as well as cell populations) are provided in Table 1. This study demonstrated that all antigens were clearly detected on cells but not on their corresponding MPs, with the exceptions of CD41 and CD61 for platelet-derived MPs. We confirmed the detection of CD41 on platelet derived MPs and the ‘‘non detection ‘‘ of CD5 on CLL B-cell derived MPs by immunogold labeling (S3 Fig).

**Table 1:** Mean fluorescence intensity ratio (MFIR) analysis on MPs by direct flow cytometry.

n = 5		Geometric Mean		
		Microparticles		
	Markers	Annexin-V +	Annexin-V -	Cells
B cell-derived MPs	CD19	1.87±0.36	1.89±0.42	12.63±0.62
	CD20	1.77±0.29	1.16±0.31	34.05±5.51
T cell-derived MPs	CD3	1.74±0.19	1.27±0.07	5.52±1.57
	CD8	1.09±+0.09	1.06±0.07	29.99±10.18
	CD5	1.64±0.17	0.96±0.05	30.93±13.4
	CD27	1.33±0.14	1.25±0.10	26.23±13.66
Monocyte-derived MPs	CD11c	1.97±0.6	1.33±0.09	13.54±0.6
	CD14	1.49±0.14	1.48±0.07	2.39±0.38
NK cell-derived MPs	CD16	1.42±0.05	1.39±0.03	42.55±5.09
	CD56	1.51±0.09	1.51±0.06	16.02±0.83
Platelet-derived MPs	CD41	10.33±3.67	2.52±0.89	6.9±2.62
	CD61	67.76±15.59	17.12±4.53	57.90±2.56

We calculated the mean of the MFIR ± SEM (Mean fluorescence intensity ratio ± standard error of the mean) for all used antibodies (n = 5). The expression of all antibodies was evaluated on 5 cell type-derived MPs, both annexin-V + and—populations and also on the original cells. These numbers demonstrated that antigens are clearly expressed on the cells but not necessary on the MPs.

### Immunophenotypic characterization by latex bead flow cytometry

**Fig 4:**
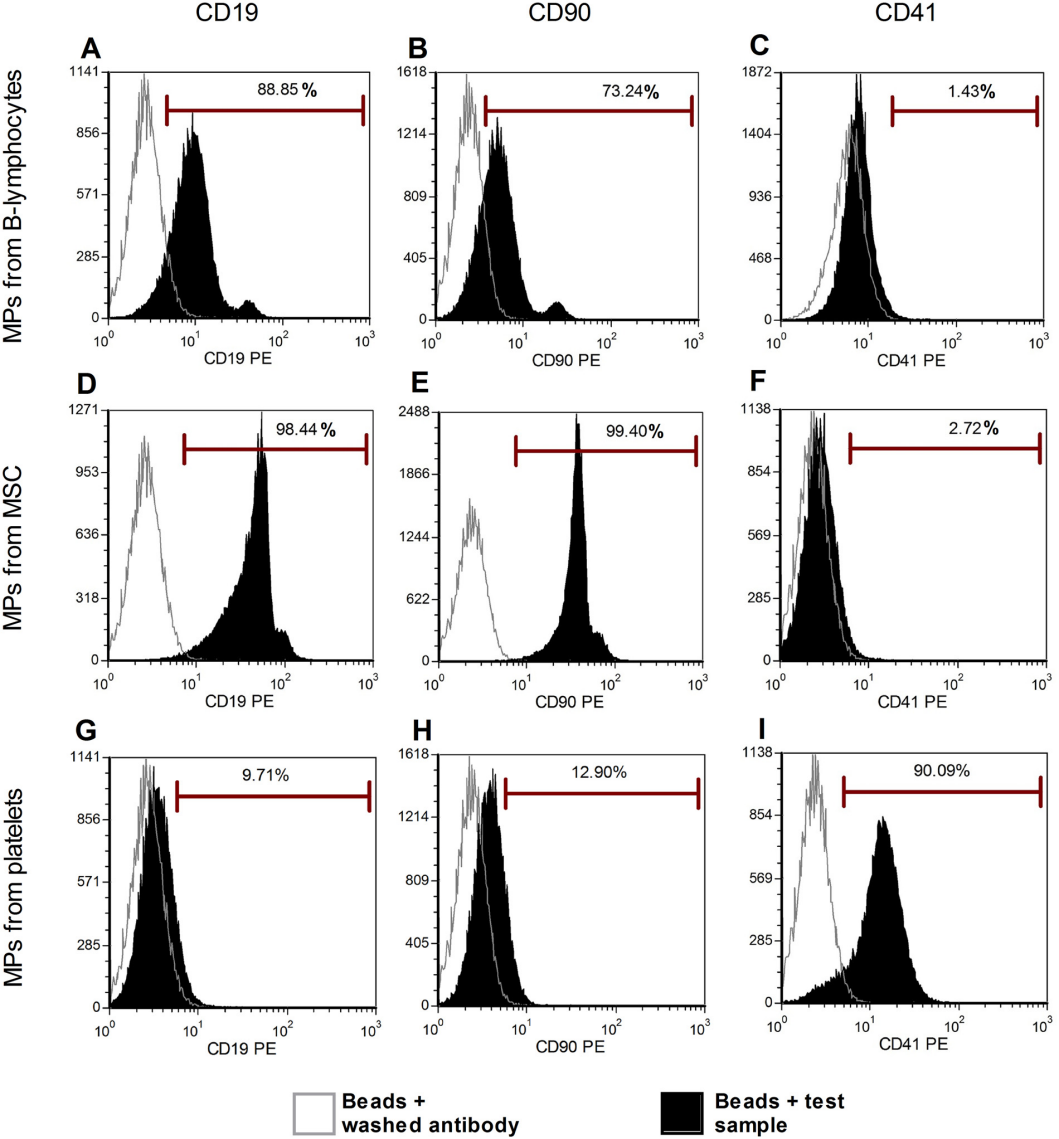
MP characterization by latex bead technique. We studied three types of cell-derived MPs with the latex bead technique: platelet-, BM-MSC- and B-cell-derived MPs. Beads with ‘‘washing antibody” were used as negative controls. Three different labels were applied to these MPs. (I) We confirmed the expression of CD41 on platelet-derived MPs, (G-H) and negative signal for CD90 and CD19. (C/F) Although CD41 expression was negative for B-cell and BM-MSC-derived MPs, (A-B, D-E) we observed CD90 and CD19 expression on both B-cells and BM-MSC-derived MPs, confirming false positive labeling.

To confirm the results obtained by direct flow cytometry, we studied MPs derived from three cell types (platelets, BM-MSC, and B-cells) with an optimized latex bead technique. Three different labels were applied to these MPs (Fig 4). Using this method, as previously described, we confirmed the expression of CD41, a platelet marker, on platelet-derived MPs (Fig 4I). This antigen was not found on the 2 other populations of MPs (Fig 4C and 4F), validating our protocol. Regarding the direct flow cytometry, positive signals were observed for some markers (CD19-PE on B cell-derived MPs, CD90 on MSC-derived MPs). However, when the different MPs were cross-labeled for these markers (CD19 on MSC-derived MPs and CD90 on B-cell-derived MPs), a positive signal was also observed, indicating that the signals were false positives.

## Discussion

Microvesicles play a crucial role in cellular interactions and have been considered a novel mechanism of cross-talk between normal and malignant cells [4]. Even if the phenotypic characterization of extracellular vesicles is well defined in the literature, there are some discrepancies concerning antigen detection. Besides, Oksvold et al. described that there is an important difference in subpopulations of exosomes derived from the same B-cell lymphoma cell line expressing their own panel of proteins [56]. In the present study, we attempted to clarify the differences found in the literature about the phenotype of several blood cell-derived MPs and we proposed a new negative control to avoid false positive detection. The expression of different antigens (CD3, 5, 8, 11c, 14, 16, 19, 20, 27, 41, 56, and 61) was investigated on B-lymphocytes, T-lymphocytes, monocytes, NK-cells, platelets, CLL B-cells and their derived MPs by two different flow cytometry techniques.

We used Megamix PLUS-beads to calibrate the Navios cytometer, several authors discussed about the impact of the refractive index difference between the beads and the MPs (n = 1.6/1.4). This point was clarified and well-argued by Robert et al, they performed differential filtrations using specific filters and proved that platelet MPs enumerated in the MP gate (0.3–1μm) were not affected by 1μm filtration, but completely depleted by 0.2μm filtration. They concluded that difference of index doesn’t affect extensively the flow cytometry forward scatter (FCM FS) results [52;60]. However, it should be noted that this subject is still under debate: some authors highlighted that flow cytometry with polystyrene/latex bead calibration can lead to an underestimation of the vesicle sizes. It should be interesting to use silica beads that have refractive index much closer than biological MVs. Even if the NAVIOS cytometer is currently one of the best cytometers [52] current flow cytometers have some limitations and are only capable to measure the ‘‘top of the iceberg”. The vesicle sizes must be considered with caution [61–65]

To keep MPs as close to their native state as possible, we did not use any activator to increase MP production, in contrast to previous work that used products such as calcium ionophore 23187, LPS and collagen [66]. Connor et al and Sims et al demonstrated that the phenotype of platelet-derived MPs can vary depending on the nature of activator used to induce their formation; notably collagen, thrombin, ADP or calcium ionophore [21;67]. Similar observations were reported by Jimenez et al for endothelial-cell-derived MPs [68] and by de Jong et al for exosomes [45]. In our study, MPs were isolated by ultracentrifugation, while filtration steps were avoided as recommended by Macey et al [36].

We considered MPs as all events within the MP gate and positive for annexin-V labeling. Although this marker is widely used in the MP field [35;52;69], the subject remains under debate. Indeed, the use of phosphatidylserine (PS) staining is not always applied to define MP populations [21;22;24;25;28]. Therefore, we analyzed both populations of MPs (annexin-V positive and negative).

Our study confirmed that CD41/CD61 expression on platelet-derived MPs can be detected by direct and latex bead flow cytometry as it was largely described in the literature [5;6]. Surprisingly, we obtained a weak positive signal for specific markers such as CD3, CD19, and CD56 for T-lymphocytes, B-lymphocytes and NK-cell-derived MPs, respectively. This labeling was shown to be a false positive using the same markers on MPs derived from different cell types (Fig 3). Moreover no other specific antigens were detected on MPs while these antigens were significantly found on the source cells. This was also confirmed with a different and more sensitive technique (immunogold labeling). We were able to detect CD41 on platelet derived MPs but no CD19/CD5 on CLL B-cells derived MPs, confirming our cytometry results (S3 Fig). Interestingly, our study showed that the expression profiles of CD41/CD61 are clearly different between annexin-V positive and negative populations. This observation was also made for other antigens by Nielsen et al and Connor et al in 2010 [21;70]. Further investigation is needed to better understand this difference.

A recent report suggested that there is a major decrease in antigen expression between cells and EVs [71]. Moreover, it has already been shown that MPs are derived from lipid rafts and we can thus hypothesize that these parts of the membrane can be characterized by differential partitioning of antigens [72–75]. Miguet et al observed by a proteomic study that while some antigens are detected on MPs, CD3, 5, and 8 for T-cells and CD19 for B-cells are not found on MPs despite being highly expressed on the original cells [76]. Our results conflict with those of authors who report detection by direct flow cytometry of CD105, CD90, and CXCR4 on MPs from mesenchymal stem cells [77] and CD3, CD14 and CD19 on MPs from the plasma of polymyositis/dermatomyositis patients [78]. In addition, Ghosh et al and Macey et al detected annexin-V-CD19+ [35] and CD15+ MPs [36], respectively. These discrepancies with our study could be explained by several factors: speed of centrifugation, choice of specific negative controls and antibodies, MP origin and the presence of specific activators in culture. Indeed, these authors analyzed MPs from cell culture or directly from plasma and they either applied centrifugation forces of 16,000 x g to 19,800 x g for 10 min to 1 h or they did not use centrifugation at all. More importantly, all of these groups used different negative controls. Baka et al used unstained samples and Kim et al, Ghosh et al, Macey et al used isotype controls. Trummer and colleagues demonstrated that using isotype negative controls can induce errors: they concluded that using flow cytometry to discriminate between positive and negative populations of MPs labeled with several antibodies could be an obstacle for their characterization [79]. All of these differences could explain the variation in results between several groups. The study of MPs is complicated by their limited surface area and relatively small number of proteins available for antibody binding. Thus, the latex bead technique has been used by several groups to facilitate the characterization of MPs by flow cytometry. Different protocols have been published [37;54;55], but we adapted the protocols of Wu et al and Mokarizadeh et al with minor modifications [39;40]. Since no significant difference was observed by using blocking reagent, we removed this step, as previously described by Oksvold et al [56]. Three different negative controls were used: beads alone without any staining, beads with MPs and isotype control and beads with MPs and a negative marker (one not present on the original cells). Isotype controls were removed from our study because they resulted in some false positive results (data not shown). Szczepanski et al, Wu et al and Mokarizadeh et al presented positive results from the first protocol, notably for CD9, CD33, CD63, CD73, and CD90 [39;40;54]. However, in our study, we demonstrated that this protocol is associated with non-specific labeling due to antibody affinity for latex beads. Indeed incubating beads with antibody in the absence of MPs resulted in an increase of background noise (data not shown). Thereafter, we designed our own protocol, by directly incubating the beads with previously labeled MPs. In this way, we avoided an increase in background signals due to non-specific binding of antibodies to beads. Using this protocol we were able to detect specific markers on platelet-derived MPs. Together, our results indicated that several previously published data sets using the latex bead technique include false positive results due to the use of inappropriate protocols or negative controls.

The goal of this study was to emphasize on the inconsistencies in literature concerning the characterization of MPs by flow cytometry and the importance of the negative control choice. We confirmed the data of Trummer and colleagues concerning the failure of classical negative control (isotype) in the MP study. Since, some antigens could be expressed by different cell types (i.e. CD5 could be found on T or B cells), several negative controls should be tested for a specific MV staining.

In conclusion, the characterization of MPs is still a challenging field requiring lot of precautions in the interpretation of the fluorescence signal. We demonstrated that isotype controls and unstained samples are not suitable for MP characterization. Therefore, we proposed using other negative markers (marker not found on the original cell) by cross-staining MPs to demonstrate the true positive labeling. We want to underline the fact that this method is applicable on cell culture supernatants specifically but not on body fluids (like urine or plasma), which are more complex samples. By this manner, non-specific antibody labeling could be subtracted out, and reliable results could be obtained.

## Supporting Information

S1 Fig CD3 titration on T cells and T cell derived MPs. (DOCX)

S2 Fig CD41 titration on platelet derived MPs. (DOCX)

S3 Fig Immunogold labeling on CLL B-cell derived and platelet derived MPs with CD5 and CD41 antibodies. (DOCX)

S1 Table Viability of cells by Trypan blue assay. (DOCX)

S2 Table All the Concentrations/Clones/Origins of antibodies used in experiments. (DOCX)

S3 Table Mean Fluorescence Intensity (MFI) and MFI Ratio (MFIR) of the CD3 labeling on T cells. These numbers demonstrated that CD3 antigen is clearly expressed on T-cells.
(DOCX)

S4 Table Mean Fluorescence Intensity (MFI) and MFI Ratio (MFIR) of the CD3 labeling on T cell derived MPs. These numbers demonstrated that CD3 antigen is not necessary expressed on T cell derived MPs.
(DOCX)
