# Supplementary figures and images for: Avoiding False Positive Antigen Detection by Flow Cytometry on Blood Cell Derived Microparticles: The Importance of an Appropriate Negative Control
Source: PLoS One. 2015 May 15;10(5):e0127209. doi: 10.1371/journal.pone.0127209 (PMC4433223; doi:10.1371/journal.pone.0127209)

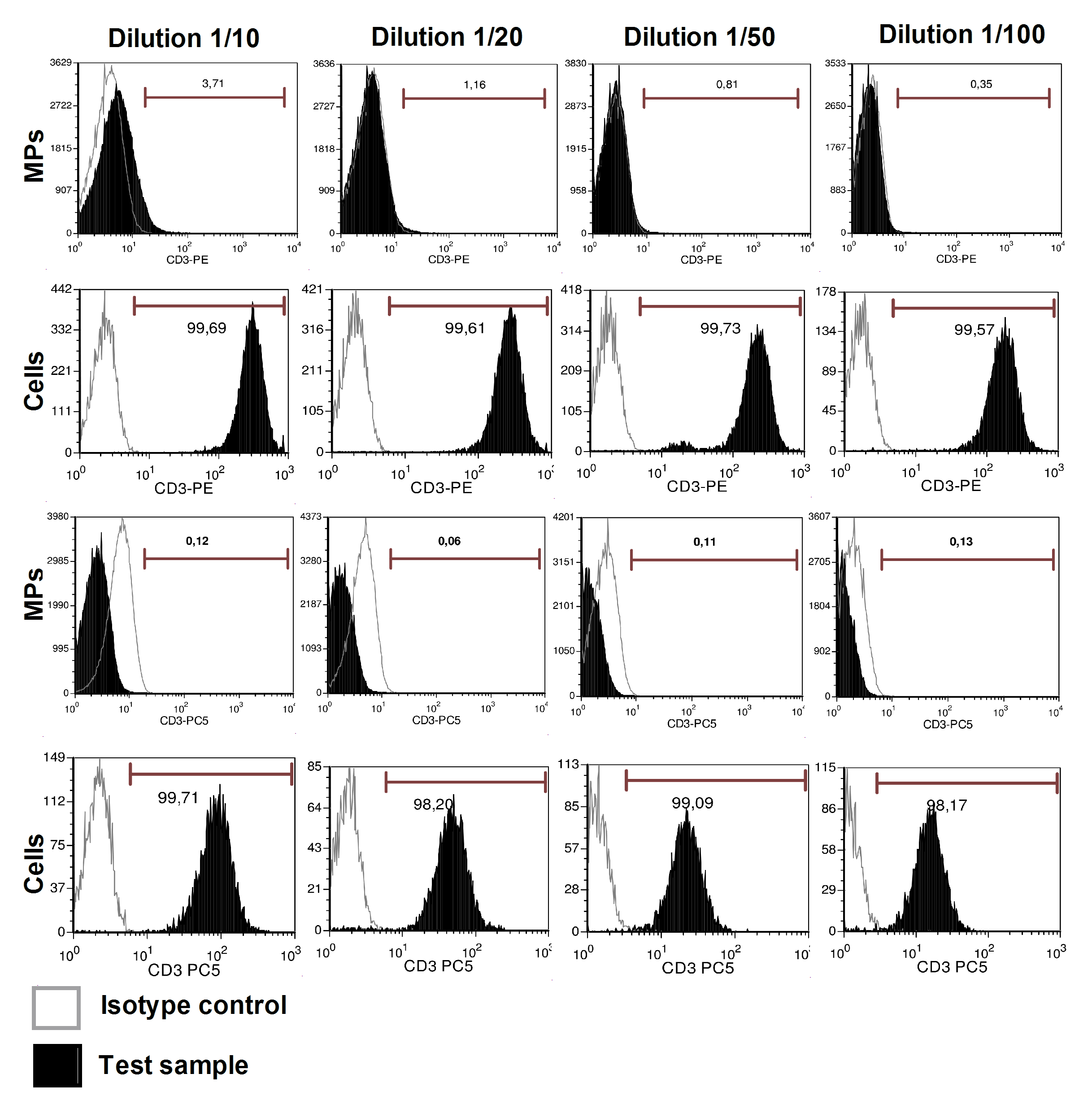


S1 Figure: CD3 titration on T cells and T cell derived MPs.

Supplement: S1 Fig — (DOCX) [file pone.0127209.s001.docx]

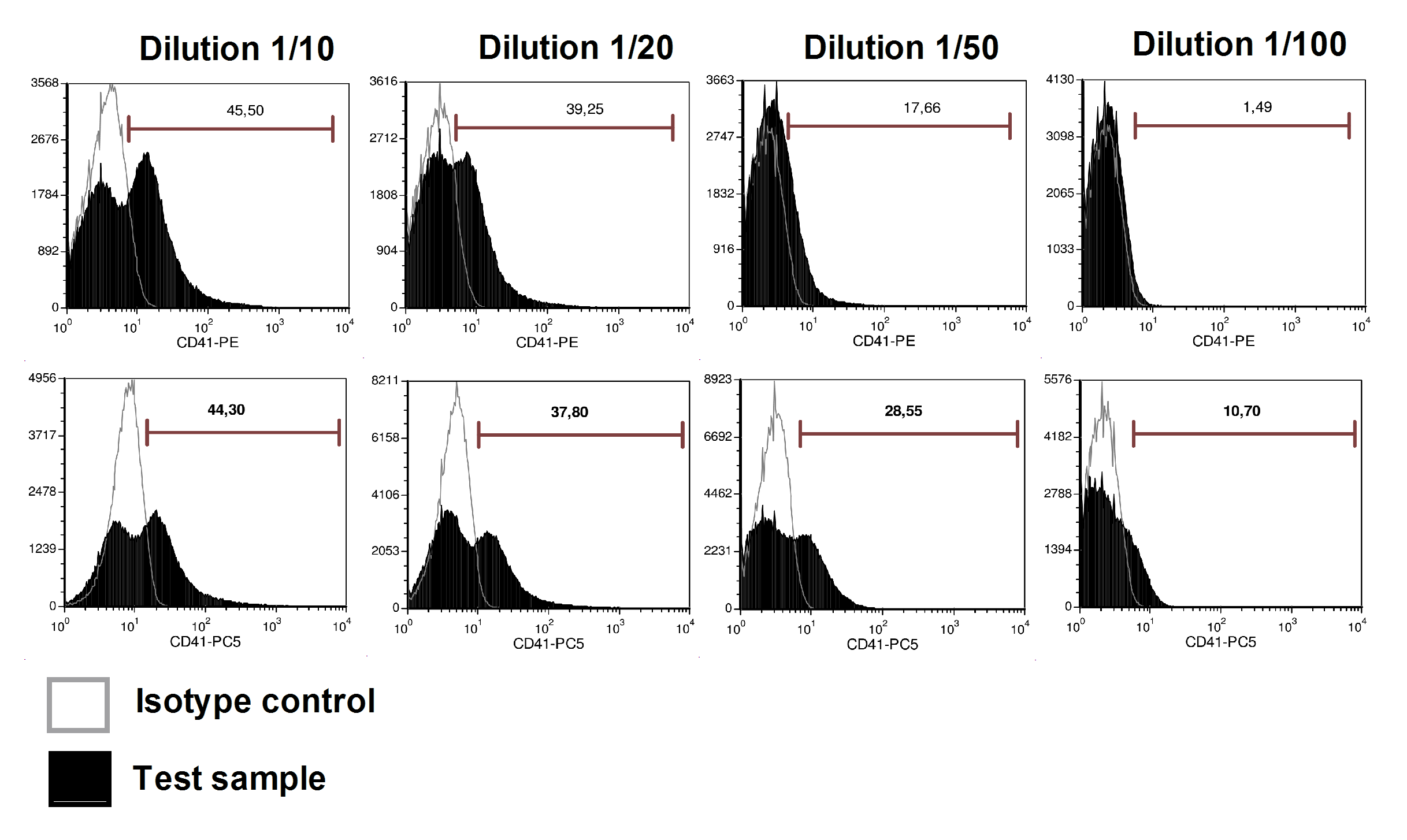


*S2 Figure: CD41 titration on platelet derived MPs.*

Supplement: S2 Fig — (DOCX) [file pone.0127209.s002.docx]
