# Supplementary material for: Avoiding False Positive Antigen Detection by Flow Cytometry on Blood Cell Derived Microparticles: The Importance of an Appropriate Negative Control
Source: PLoS One. 2015 May 15;10(5):e0127209. doi: 10.1371/journal.pone.0127209 (PMC4433223; doi:10.1371/journal.pone.0127209)
Supplement: S3 Fig — (DOCX) [file pone.0127209.s003.docx]

*
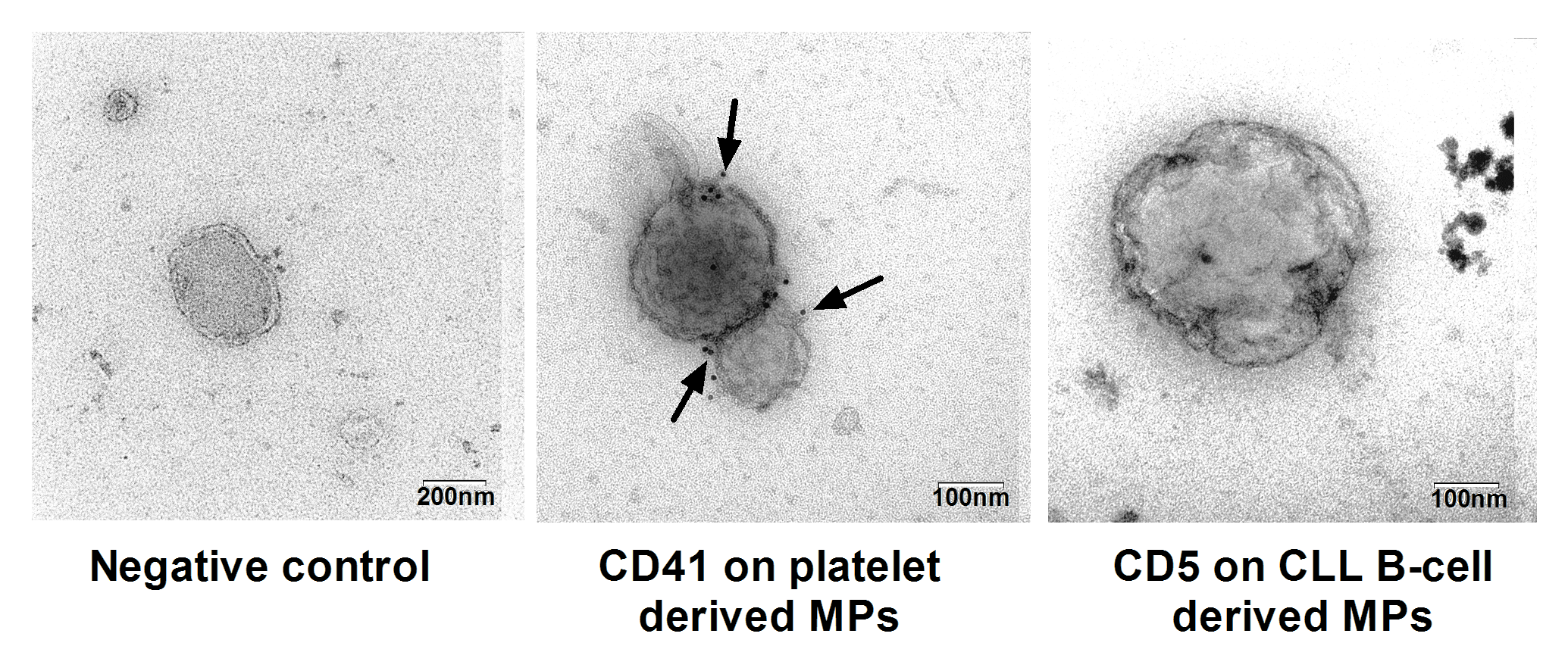
S3 Figure: Immunogold labeling on CLL B-cell derived and platelet derived MPs with CD5 and CD41 antibodies.*
