# Supplementary material for: Avoiding False Positive Antigen Detection by Flow Cytometry on Blood Cell Derived Microparticles: The Importance of an Appropriate Negative Control
Source: PLoS One. 2015 May 15;10(5):e0127209. doi: 10.1371/journal.pone.0127209 (PMC4433223; doi:10.1371/journal.pone.0127209)
Supplement: S1 Table — (DOCX) [file pone.0127209.s004.docx]

***S1 Table :*** Viability of cells by Trypan blue assay

| ***Cell type*** | ***Viability*** |
| --- | --- |
| T cells | 96.07% ±1.1 |
| B cells | 88.6% ±2.26 |
| Monocytes | 77.33% ±5.84 |
| NK cells | 79.53% ±1.98 |
| CLL-B cells | 94.07% ± 0.52 |
