# Supplementary material for: Avoiding False Positive Antigen Detection by Flow Cytometry on Blood Cell Derived Microparticles: The Importance of an Appropriate Negative Control
Source: PLoS One. 2015 May 15;10(5):e0127209. doi: 10.1371/journal.pone.0127209 (PMC4433223; doi:10.1371/journal.pone.0127209)
Supplement: S2 Table — (DOCX) [file pone.0127209.s005.docx]

***S2 Table:*** *A*ll the Concentrations/Clones/Origins of antibodies used in experiments

| Antibodies | Concentration (µg/ml) | Clone | Origin |
| --- | --- | --- | --- |
| CD3 PE | 11µg | BW264/56 | Miltenyi |
| CD3PC5 | 3.5µg | UCHT1 | BDbiosciences |
| CD41PE | 200µg | P2 | Beckman |
| CD41PC5 | 6µg | HIP8 | Biolegend |
| CD19PE | 22µg | LT19 | Miltenyi |
| CD14PE | 8.25µg | TÜK4 | Miltenyi |
| CD61PE | 33µg | Y2/51 | Miltenyi |
| CD19PC5 | 3µg | HIB19 | BDbiosciences |
| CD20PE | 3µg | 2H7 | BDbiosciences |
| CD11cPE | 3µg | B-ly6 | BDbiosciences |
| CD16PE | 200µg | 3G8 | Beckman |
| CD56PC5 | 200µg | HLDA6 | Beckman |
| CD5PE | 200µg | BL1a | Beckman |
| CD8PC5 | 20µg | DK25 | Dako |
| CD27 PercP | 200µg | 323 | Biolegend |
| Isotype PE | 12.5µg | MOPC-21 | BDbiosciences |
| Isotype PC5 | 6.25µg | MOPC-21 | BDbiosciences |
| Isotype APC | 12.5µg | MOPC-21 | BDbiosciences |
| Isotype PE | 8µg | S4310 | Miltenyi |
