# Supplementary material for: Avoiding False Positive Antigen Detection by Flow Cytometry on Blood Cell Derived Microparticles: The Importance of an Appropriate Negative Control
Source: PLoS One. 2015 May 15;10(5):e0127209. doi: 10.1371/journal.pone.0127209 (PMC4433223; doi:10.1371/journal.pone.0127209)
Supplement: S3 Table — These numbers demonstrated that CD3 antigen is clearly expressed on T-cells. (DOCX) [file pone.0127209.s006.docx]

*S3 Table:* Mean Fluorescence Intensity (MFI) and MFI Ratio (MFIR) of the CD3 labeling on T cells. These numbers demonstrated that CD3 antigen is clearly expressed on T-cells.

| antibodies | dilution | MFI | MFIR |
| --- | --- | --- | --- |
| ISO PE | dil1/10 | 2.25 |  |
| ISO PE | dil1/20 | 2.02 |  |
| ISO PE | dil1/50 | 1.78 |  |
| ISO PE | dil1/100 | 1.71 |  |
| CD3PE | dil1/10 | 289.81 | 128.8 |
| CD3PE | dil1/20 | 258.32 | 127.9 |
| CD3PE | dil1/50 | 206.74 | 116.1 |
| CD3PE | dil1/100 | 166.92 | 97.6 |
| ISO PC5 | dil1/10 | 2.1 |  |
| ISO PC5 | dil1/20 | 1.7 |  |
| ISO PC5 | dil1/50 | 1.36 |  |
| ISO PC5 | dil1/100 | 1.24 |  |
| CD3PC5 | dil1/10 | 84.32 | 40.2 |
| CD3PC5 | dil1/20 | 46.89 | 27.6 |
| CD3PC5 | dil1/50 | 21.49 | 15.8 |
| CD3PC5 | dil1/100 | 15.38 | 12.4 |
