# Supplementary material for: Avoiding False Positive Antigen Detection by Flow Cytometry on Blood Cell Derived Microparticles: The Importance of an Appropriate Negative Control
Source: PLoS One. 2015 May 15;10(5):e0127209. doi: 10.1371/journal.pone.0127209 (PMC4433223; doi:10.1371/journal.pone.0127209)
Supplement: S4 Table — These numbers demonstrated that CD3 antigen is not necessary expressed on T cell derived MPs. (DOCX) [file pone.0127209.s007.docx]

*S4 Table:* Mean Fluorescence Intensity (MFI) and MFI Ratio (MFIR) of the CD3 labeling on T cell derived MPs. These numbers demonstrated that CD3 antigen is not necessary expressed on T cell derived MPs.

| anticorps | dilution | MFI | MFIR |
| --- | --- | --- | --- |
| ISO PE | dil1/10 | 3.04 |  |
| ISO PE | dil1/20 | 2.85 |  |
| ISO PE | dil1/50 | 2.06 |  |
| ISO PE | dil1/100 | 1.91 |  |
| CD3PE | dil1/10 | 3.57 | 1.2 |
| CD3PE | dil1/20 | 2.78 | 1.0 |
| CD3PE | dil1/50 | 2.19 | 1.1 |
| CD3PE | dil1/100 | 1.84 | 1.0 |
| ISO PC5 | dil1/10 | 6.0 |  |
| ISO PC5 | dil1/20 | 4.0 |  |
| ISO PC5 | dil1/50 | 2.4 |  |
| ISO PC5 | dil1/100 | 1.7 |  |
| CD3PC5 | dil1/10 | 2.2 | 0.4 |
| CD3PC5 | dil1/20 | 1.5 | 0.4 |
| CD3PC5 | dil1/50 | 1.3 | 0.6 |
| CD3PC5 | dil1/100 | 1.2 | 0.7 |
